# Preoperative identification of low-risk medullary thyroid carcinoma: potential application to reduce total thyroidectomy

**DOI:** 10.1038/s41598-023-42907-3

**Published:** 2023-09-20

**Authors:** Hyunju Park, Hyun Jin Ryu, Jung Heo, Man Ki Chung, Young Ik Son, Jung-Han Kim, Soo Yeon Hahn, Jung Hee Shin, Young Lyun Oh, Sun Wook Kim, Jae Hoon Chung, Jee Soo Kim, Tae Hyuk Kim

**Affiliations:** 1 Department of Internal Medicine, CHA Bundang Medical Center, CHA University School of Medicine, Seongnam, Korea; 2 Division of Endocrinology and Metabolism, Department of Medicine, Thyroid Center, Samsung Medical Center, Sungkyunkwan University School of Medicine, 115 Irwon-Ro, Gangnam-Gu, Seoul, 06355 Korea; 3 Department of Internal Medicine, Yonsei University Wonju College of Medicine, Wonju-si, Gangwon-do Korea; 4 Department of Otorhinolaryngology-Head and Neck Surgery, Samsung Medical Center, Sungkyunkwan University School of Medicine, Seoul, Korea; 5 Division of Breast and Endocrine Surgery, Department of Surgery, Samsung Medical Center, Sungkyunkwan University School of Medicine, 81 Irwon-Ro, Gangnam-Gu, Seoul, 06351 Korea; 6 Department of Radiology, Samsung Medical Center, Sungkyunkwan University School of Medicine, Seoul, Korea; 7 Department of Pathology and Translational Genomics, Samsung Medical Center, Sungkyunkwan University School of Medicine, Seoul, Korea

**Keywords:** Endocrinology, Risk factors

## Abstract

Current guidelines recommend total thyroidectomy with central lymph node dissection (CND) for patients with medullary thyroid carcinoma (MTC). This study aimed to identify low-risk MTC patients who may be candidates for lobectomy. We retrospectively reviewed MTC patients who underwent primary surgery at a tertiary referral center from 1998 to 2019. Eighty-five MTC patients were enrolled, excluding patients with primary tumor size > 2.0 cm. Among them, one (1.2%) patient had bilateral tumors. During a median follow-up of 84 months, 12 of the 85 patients experienced structural recurrence. 13 patients had occult lymph node metastasis, and structural recurrence occurred in 2 patients. Factors that significantly affected disease-free survival were clinical N stage (cN0 vs. cN1, log-rank *P* < 0.001), pathological N stage (pN0 vs. pN1, *P* < 0.001), and preoperative calcitonin levels (≤ 250 vs. > 250 pg/mL, *P* = 0.017). After categorizing patients into four groups, patients with preoperative calcitonin levels > 250 pg/mL and cN1 or pN1 had a significantly worse prognosis. Patients with a primary tumor size of 2 cm or less, cN0, and preoperative calcitonin of 250 pg/mL or less can be classified as low-risk MTC patients. We used preoperative clinical information to identify low-risk MTC patients. Lobectomy with prophylactic CND may be a potential therapeutic approach.

## Introduction

Medullary thyroid carcinoma (MTC) is an uncommon malignancy that derives from the parafollicular C cells of the thyroid. MTC occurs in either a sporadic form or hereditary and familial form, accounting for approximately 75% and 25% of cases, respectively^1,2^. The cancer-related mortality in MTC has been reported as high as 15%^3,4^; thus, MTC has a worse prognosis than that of differentiated thyroid carcinoma (DTC).

During the past three decades, the clinicopathological features and clinical outcomes of thyroid cancer have changed over time^5–7^. Due to the development of high-resolution neck ultrasonography (US), thyroid carcinoma can be diagnosed at an early stage, and diagnosis of MTC has improved significantly. Previous studies reported that primary tumor size had significantly decreased at the time of diagnosis; the proportions of tumors ≤ 1 cm and pathologic N0 patients have increased over time^7–9^. Since baseline clinicopathological characteristics have changed, disease-free survival (DFS) of MTC has improved. Randle et al. reported that 5-year DFS significantly improved from 86 to 89%. However, 5-year DFS was not significantly different for those with local disease^8^. Interestingly, Roman et al. also reported that overall survival was not significantly different for patients with localized MTC who underwent lobectomy and total thyroidectomy^10^. Although guidelines suggest that total thyroidectomy with central lymph node dissection (CND) is the standard treatment for MTC^11–13^, lobectomy with prophylactic CND might be sufficient in selected patients.

Almost all hereditary MTC cases are bilateral, but fewer than 10% of sporadic MTC patients have bilateral disease^14^. Recently, overtreatment of indolent cancers raised a problem, and attempts to reduce over-diagnosis or over-treatment have occurred. Optimal management can reduce morbidity, improve treatment effectiveness and patient quality of life, and increase quality-adjusted life years^15,16^. The purpose of this study was to identify low-risk MTC patients who are potential candidates for lobectomy with prophylactic CND. This study provides evidence for the need for a paradigm shift in treatment of localized MTC.

## Results

### Baseline characteristics

The clinicopathological characteristics of the 85 MTC patients are presented in Table 1. The mean (SD) age was 52.9 (10.57) years, and 56 (65.9%) were female. MTC was diagnosed by preoperative fine-needle aspiration in 69 (81.2%) patients. Bilaterality was observed in only one (1.2%) patient. Most enrolled patients (91.8%) underwent more than total thyroidectomy with CND. Only three patients underwent less than total thyroidectomy, and four patients underwent total thyroidectomy without lymph node dissection. The median (IQR) size of the primary tumor was 1.0 (0.7–1.5) cm, and the primary tumor size was ≤ 1 cm in 45 (52.9%) patients. The distribution of cN0, cN1a, and cN1b classifications was 65 (76.5%), 2 (2.4%), and 18 (21.2%), respectively. The distribution of pNx/pN0, pN1a, and pN1b classifications was 54 (63.5%), 12 (14.1%), and 19 (22.4%), respectively. Forty-eight (57.8%) patients had preoperative serum calcitonin ≤ 250 pg/mL, and the disease recurred in 12 (14.1%) patients. All patients tested for RET mutations had wild-type results. The 29 patients not tested for RET mutations were clinically diagnosed with sporadic MTC.

**Table 1:** Baseline characteristics.

	Number (%)
Age, years (mean, SD)	52.9 (10.57)
Gender (n, %)	
Female	56 (65.9)
Male	29 (34.1)
Fine-needle aspiration results (n, %)	
Benign	9 (10.6)
Suspicious of MTC	5 (5.9)
MTC	69 (81.2)
Thyroid carcinoma except MTC	2 (2.4)
Bilaterality (n, %)	
No	84 (98.8)
Yes	1 (1.2)
Extent of surgery (n, %)	
Lobectomy/isthmectomy with or without lymph node dissection	3 (3.5)
Total thyroidectomy without lymph node dissection	4 (4.7)
Total thyroidectomy with CND	34 (40.0)
Total thyroidectomy with CND with ipsilateral lateral lymph node dissection	40 (47.1)
Total thyroidectomy with CND with contralateral lateral lymph node dissection	4 (4.7)
Primary tumor size, cm (median, IQR)	1.0 (0.7–1.5)
≤ 1.0 cm	45 (52.9)
> 1 cm and ≤ 2 cm	40 (47.1)
Regional lymph node metastasis, clinical (n, %)	
cN0	65 (76.5)
cN1a	2 (2.4)
cN1b	18 (21.2)
Regional lymph node metastasis, pathological (n, %)	
pNx/0	54 (63.5)
pN1a	12 (14.1)
pN1b	19 (22.4)
Preoperative calcitonin, pg/mL (mean, SD)^a^	
≤ 250	48 (57.8)
> 250	35 (42.2)
Preoperative CEA, ng/mL (median, IQR)^b^	3.9 (1.6–17.0)
RET testing results (n, %)	
RET mutations	0 (0.0)
Wild-type	56 (65.9)
Not assessed	29 (34.1)
Disease recurrence (n, %)	12 (14.1)

*SD* standard deviation, *n* number of patients, *MTC* medullary thyroid carcinoma, *CND* central lymph node dissection, *IQR* interquartile range, *CEA* carcinoembryonic antigen.

^a^Two patients did not have preoperative serum calcitonin results.

^b^Thirty-four patients did not have preoperative serum CEA results.

### Distribution of clinical and pathological N stages

Table 2 shows the pathological results of preoperative US and/or CT for the detection of metastatic LNs. Occult LN metastases which are defined as pathologically confirmed metastatic LN without suspicion of metastasis on the preoperative image were identified in 13 of 65 (20.0%) patients Among them, nine were pN1a and four were pN1b. One patient classified as cN1a and three patients as cN1b had false positive results on preoperative US and/or CT. Of the 13 patients with occult LN metastasis, two experienced structural recurrences.

**Table 2:** Preoperative image versus histopathologic findings.

Histopathological finding	Preoperative image, number (%)			
	cN0	cN1a	cN1b	Total
pN0/Nx	52 (80.0)	1 (50.0)	1 (5.6)	54
pN1a	**9 (13.8)**	1 (50.0)	2 (11.1)	12
pN1b	**4 (6.2)**	0 (0.0)	15 (83.3)	19
Total	65	2	18	85

Occult lymph node metastases are in bold.

### Disease-free survival according to clinicopathological parameters

**Figure 1:**
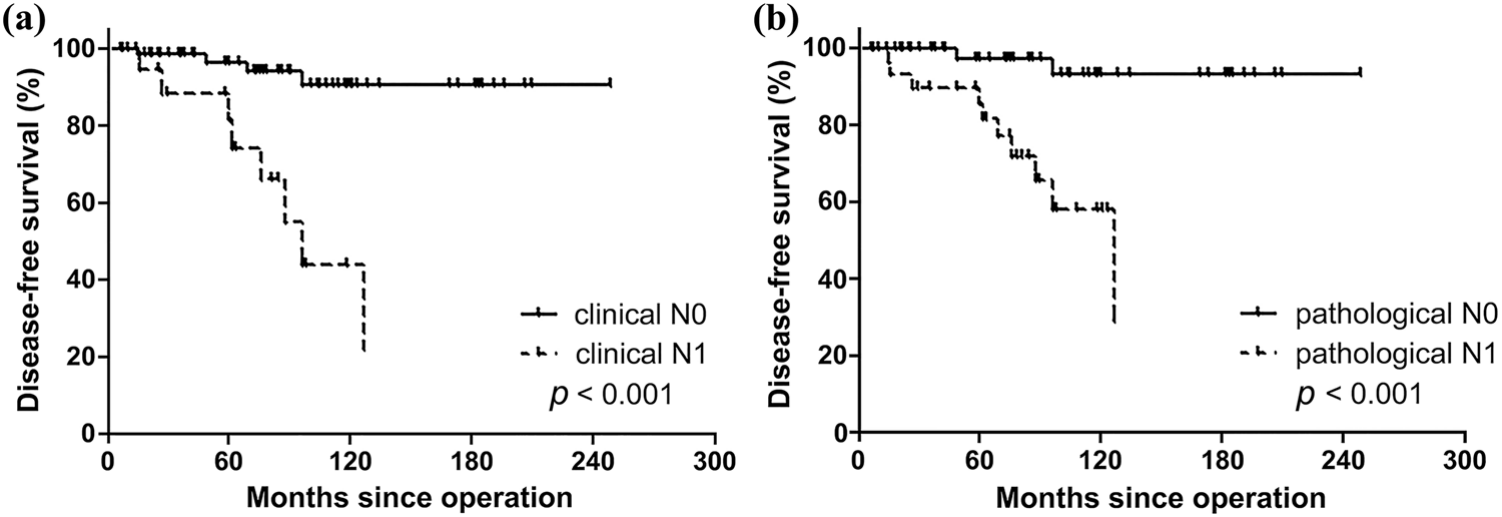
Disease-free survival according to nodal staging: (**a**) clinical N staging and (**b**) pathological N staging.

The Kaplan–Meier survival curve for DFS is shown in Fig. 1. According to the clinical N stage, four of 65 cN0 patients and eight of 20 cN1a/b patients experienced structural recurrence. DFS was significantly different between cN0 and cN1a/b groups (log-rank *P* < 0.001) (Fig. 1a). When comparing DFS according to pathological N stage, a similar result was observed (Fig. 1b). Two of 54 pNx/0 and 10 of 31 cN1a/b patients experienced structural recurrences. DFS was significantly different between pNx/0 and pN1a/b groups (log-rank *P* < 0.001). Among the 12 patients with structural recurrence, two had occult LN metastasis, and 10 had consistent results with preoperative US and/or CT findings.

**Figure 2:**
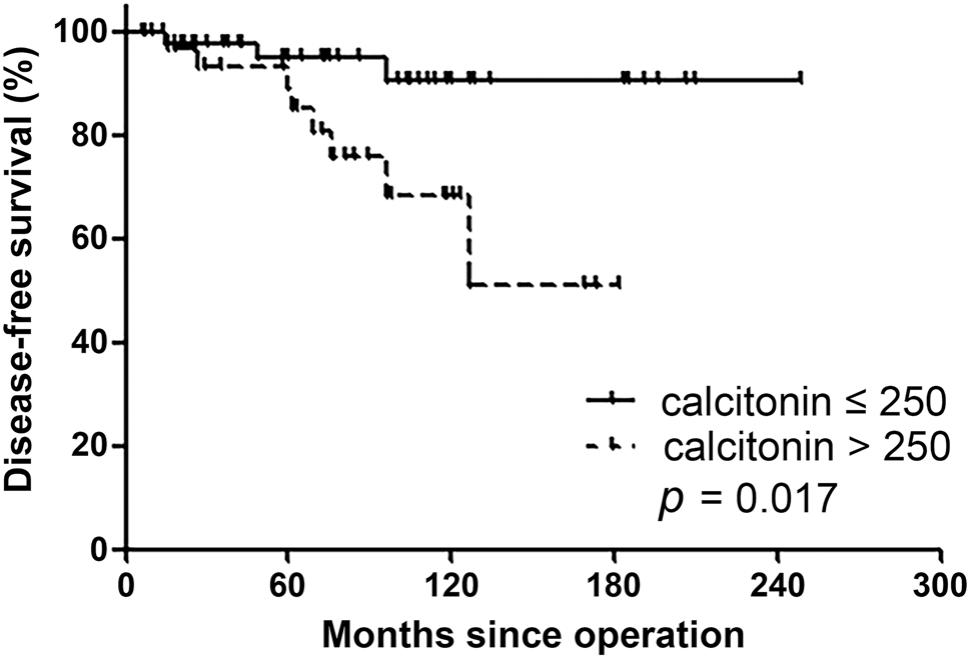
Disease-free survival according to preoperative serum calcitonin level.

We compared DFS according to preoperative serum calcitonin levels (Fig. 2). Of 85 patients, 83 had preoperative serum calcitonin results. We used serum calcitonin 250 pg/mL as the cut-off level. Three of 48 patients with ≤ 250 pg/mL and 8 of 35 patients with > 250 pg/mL experienced disease recurrence (log-rank *P* = 0.017).

**Figure 3:**
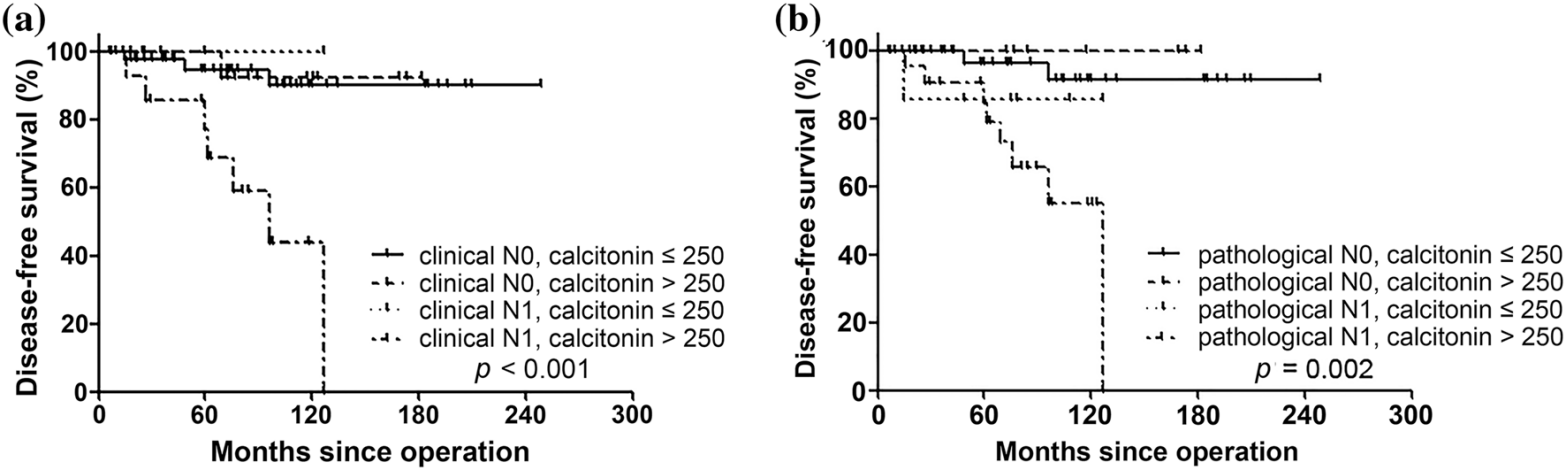
Disease-free survival in four groups categorized based on nodal staging and preoperative serum calcitonin level. (**a**) clinical N staging with preoperative serum calcitonin levels and (**b**) pathological N staging with preoperative serum calcitonin levels.

Using the above two criteria, we categorized patients into four groups (Fig. 3a, Supplementary Table 1). Patients with cN0 had excellent clinical outcomes even if their serum calcitonin was > 250 pg/mL. DFS was significantly poorer in patients with clinical or pathological LN metastasis and serum calcitonin > 250 pg/mL. Similar results were seen when patients were categorized into four groups based on pathologic N stage and preoperative serum calcitonin levels (Fig. 3b).

The results of the subsequent Cox proportional hazards analysis are shown in Table 3. Multivariate analysis was performed using clinical and pathologic categories separately. In model 1, cN1 with calcitonin > 250 pg/mL was associated with significantly worse disease-free survival on multivariate analysis (hazard ratio [HR] 9.06, 95% confidence interval [CI] 2.30–35.62, *P* = 0.002). In model 2, pN1 with calcitonin > 250 pg/mL was associated with worse disease-free survival on multivariate analysis (HR 19.75, 95% CI 2.40–162.77, *P* = 0.006).

**Table 3:** Univariate and multivariate Cox regression analysis of disease-free survival.

	Univariate		Multivariate			
			Model 1^a^		Model 2^b^	
	HR (95% CI)	*P* value	HR (95% CI)	*P* value	HR (95% CI)	*P* value
Age, years	0.99 (0.93–1.05)	0.776				
Gender						
Male	Reference					
Female	0.63 (0.20–2.01)	0.438				
Initial CND						
No	Reference					
Yes	1.33 (0.17–10.43)	0.788				
Regional lymph node metastasis, clinical						
cN0	Reference					
cN1a	16.47 (1.73–156.63)	0.015				
cN1b	7.80 (2.27–26.77)	0.001				
Regional lymph node metastasis, pathological						
pNx/0	Reference					
pN1a	7.75 (1.27–47.26)	0.026				
pN1b	12.64 (2.56–62.34)	0.002				
Primary tumor size, cm						
≤ 1.0 cm	Reference					
> 1 cm and ≤ 2 cm	0.65 (0.20–2.27)	0.485				
Preoperative calcitonin, pg/mL						
≤ 250	Reference					
> 250	4.42 (1.17–16.76)	0.029				
Clinical category						
cN0, CT ≤ 250 pg/mL	Reference		Reference			
cN0, CT > 250 pg/mL	0.89 (0.09–8.60)	0.922	0.89 (0.09–8.60)	0.922		
cN1, CT ≤ 250 pg/mL	N/A		N/A			
cN1, CT > 250 pg/mL	9.06 (2.30–35.62)	0.002	9.06 (2.30–35.62)	0.002		
Pathological category						
pN0, CT ≤ 250 pg/mL	Reference	0.981			Reference	
pN0, CT > 250 pg/mL	N/A				N/A	
pN1, CT ≤ 250 pg/mL	3.02 (0.27–33.43)	0.368			5.61 (0.34–91.89)	0.227
pN1, CT > 250 pg/mL	9.69 (1.98–47.43)	0.005			19.75 (2.40–162.77)	0.006

*HR* hazard ratio, *CI* confidential interval, *CND* central lymph node dissection, *CT* calcitonin.

^a^Model 1: age, gender, initial CND, primary tumor size, category with clinical category.

^b^Model 2: age, gender, initial CND, primary tumor size, category with pathological category.

## Discussion

This study retrospectively reviewed MTC patients with a primary tumor size of 2.0 cm or less (T1) during a median (IQR) follow-up of 84 (42.5–120) months. Preoperative clinical information, calcitonin levels, and final pathologic findings were evaluated to identify low-risk MTC patients. In univariate analysis, clinical N1, pathologic N1, and preoperative serum calcitonin levels were significant factors for disease-free survival. We categorized patients into four groups according to clinical or pathologic N stage, and preoperative serum calcitonin levels. In multivariate analysis, cN1 with calcitonin levels above 250 pg/mL had a significantly worse prognosis, and we obtained similar results when analyzed by pathologic N stage. Based on these results, we can assume that detailed preoperative information can predict disease-free survival similar to postoperative outcomes. Patients with a primary tumor size of 2.0 cm or less in clinical N0 stage with a preoperative serum calcitonin level of 250 pg/mL or less may have low-risk MTC. In this group, 5-year DFS and 10-year DFS are 94.7% and 90.2%, respectively. Lobectomy with prophylactic CND may be an option for optimal surgical coverage.

MTC guidelines agree that the standard treatment for both sporadic and hereditary MTC is total thyroidectomy with CND^11–13^. The rationale for total thyroidectomy is the bilaterality of MTC; the hereditary nature of the MTC might not be evident before surgery^17,18^. However, only 5.6% of sporadic MTCs are bilateral according to an international multicenter study^14^. Miyauchi et al. reported clinical features of sporadic MTC based on germline RET mutation results. None of the 30 patients negative for germline RET mutations had bilateral MTC, whereas 5 of 6 patients positive for germline RET mutations had bilateral MTC^19^. Sporadic MTC tends to be unicentric^20^. In this study, all patients tested for RET mutations were wild-type, and those not tested for RET mutations were presumed to have clinically sporadic MTC. One patient had bilateral MTC, and this patient achieved a biochemical remission. Miyauchi et al. further suggested that lobectomy with modified radical neck dissection is appropriate for sporadic MTC based on their results from a prospective trial with 15 MTC patients^21^. Considering the development of diagnostic and genetic screening tools and recent data, we should reconsider the optimal surgical extent in MTC patients.

There was an increase in the proportion of patients with primary tumor size ≤ 1 cm and pN0 and a decrease in the proportion of patients with extrathyroidal extension (ETE) over time^7,8^. The widespread use of neck US can explain the improved identification of MTCs at an early, like papillary thyroid carcinoma^5^. Interestingly, 5-year DFS was not significantly different in local diseases, despite DFS being significantly improved in all patients. Five-year DFS based on time interval was 98%, 98%, and 99% in 1983 to 1992, 1993 to 2002, and 2003 to 2012, respectively^8^. This highlights the possibility that some low-risk patients might have undergone excessively extensive treatment to adhere to current guidelines. In the early 1900s, radical mastectomy was a standard treatment for breast cancer. However, radical mastectomy is no longer necessary because of early cancer detection with the advent of mammography. Now, breast-conserving surgery seems equally effective in most patients with fewer postoperative complications. Radical mastectomy might be helpful in the early 1900s when patients were initially diagnosed with advanced stage, but it is no longer appropriate when patients’ baseline characteristics were changed due to the early diagnosis over time^22^. In fact, the proportion of MTC patients undergoing lobectomy in Korea between 2004 and 2016 was 35%^23^. Interestingly, the rate of completion thyroidectomy was only 3.3%. This might be related to a change in baseline characteristics of MTC patients. A completion thyroidectomy may not have been necessary for some low-risk patients who underwent lobectomy. If there is no significant difference in oncologic outcomes between lobectomy and total thyroidectomy, completion surgery may not be necessary for MTC, as radioactive iodine therapy for remnant ablation is not required in cases of MTC. Indeed, postoperative quality of life and surgical comorbidity are also important. Lobectomy patients might not need to take lifelong thyroid hormone replacement, we should reconsider whether total thyroidectomy is truly necessary in low-risk MTC patients^16,24^. A more conservative surgical approach might be sufficient in these patients.

Roman et al. reported that extensive thyroid surgery was not associated with overall survival in MTC patients with localized tumors. The strongest independent predictor for survival was stage at diagnosis^10,13^. Kwon et al. also reported that pN1b tumor status was a significant factor for DFS. Furthermore, DFS was similar between those with pN0 and pN1a tumors^7^. Romen et al. found that extent of surgery was not significant in localized MTC patients but they could not provide clinical information or degree of surgical detail such as CND, unilateral or bilateral lateral lymph node dissection because of the limitation of the SEER data. In this study, we attempted to stratify the candidates for limited surgery using preoperative clinical information. Stratified low-risk MTC patients could be candidates for lobectomy.

In general, the stage at diagnosis including primary tumor size, the extent of lymph node metastasis, and the presence of distant metastasis are significant factors for the prognosis of MTC^3,10,25–27^. Adam et al. proposed a new TNM grouping that mainly downgrades T2, T4, and N1 patients. Advanced T and N1b stage are not equivalent to M1. Patients with distant metastasis showed significantly poorer prognosis than locally advanced patients^28,29^. Thus, we excluded patients with distant metastasis. Kuo et al. reported that primary tumor size larger than 2 cm is an independent risk factor for repeat surgery, and Machens et al. reported that DFS and cancer-specific survival are significantly better when the primary tumor is ≤ 2 cm^25,27^. In our study cohort, DFS showed a significant difference between 85 patients with primary tumor size ≤ 2 cm and 44 patients with primary tumor size > 2 cm (log-rank *P* = 0.015). These 44 patients were excluded due to their primary tumor size. In addition, primary tumor size ≤ 2 cm is one of the absolute indications for lobectomy in differentiated thyroid carcinoma^30,31^. Based on those results, we chose a primary tumor size ≤ 2 cm as our arbitrary threshold. The presence of lymph node metastasis at diagnosis is an important independent prognostic factor^3,25,27,32,33^. However, prognostic significance of lymph node metastasis can differ according to the size and number of involved lymph nodes. In DTC, microscopic N1 disease has a much lower risk of recurrence^34^. A similar pattern was seen in MTC patients. Only two of 13 patients (15.4%) with an occult LN had disease recurrence, whereas eight of 20 patients (40%) at cN1 stage at diagnosis had disease recurrence. The clinical significance of an occult LN seems to be minimal. Thus, cN0 patients could be low-risk MTC.

Preoperative calcitonin levels can predict the prognosis of the disease^35,36^. Since the preoperative calcitonin level can reflect the extent of the disease^37,38^, we considered this factor when stratifying patients in this study. According to the ATA guidelines for MTC, serum calcitonin levels should be considered when deciding the surgical extent in patients with no evidence of regional LN metastases on preoperative US^11^. Machens et al. reported that 20 pg/mL and 200 pg/mL can be used as cut-off values for prophylactic ipsilateral lateral lymph node dissection and contralateral lateral lymph node dissection, respectively^37^. However, controversy exists over the necessity for prophylactic lateral lymph node dissection for those LNs considered normal on preoperative US. Considering that serum calcitonin reflects both extent of LN metastasis and primary tumor size, a 20 pg/mL cut-off for prophylactic ipsilateral lateral LN dissection in patients with apparently normal LNs on US is too low. Previous studies reported that serum calcitonin levels of 250–309 pg/mL were associated with disease recurrence^35,36^. Based on these previous reports, we chose serum calcitonin ≤ 250 pg/mL as an arbitrary cut-off for low-risk MTC patients, minimizing the risk of recurrence. According to the result of this study, the effect of serum calcitonin > 250 pg/mL on prognosis was minimal in patients with no evidence of LN metastasis on preoperative US.

This study had several limitations. First, this study could not directly compare lobectomy and total thyroidectomy. Current guidelines suggest total thyroidectomy as the standard treatment and most of the enrolled patients underwent more than total thyroidectomy with CND. Thus, surgical intervention might have affected the recurrence rate. To overcome this limitation, further prospective studies will be needed. Second, this study was conducted at a single tertiary referral center and focused on primary tumor size of 2.0 cm or less (T1). As a result, a relatively small number of MTC patients were included. Considering the low incidence of MTC, further multicenter studies are needed. In addition, the follow-up period was relatively short, so we were unable to evaluate cancer-specific survival. However, previous reports lacked detailed clinical and pathologic information due to limitations in SEER or Korean National Health Insurance Service database^8,10,23^. In contrast, we were able to obtain detailed clinical information before surgery, including preoperative calcitonin levels, preoperative imaging, and postoperative pathologic data.

## Conclusion

In conclusion, patients with primary tumors ≤ 2 cm, cN0, and preoperative serum calcitonin ≤ 250 pg/mL are at a low-risk of MTC. Total thyroidectomy may not be necessary in these patients. Lobectomy with prophylactic CND may be a potential therapeutic approach.

## Materials and methods

### Study population

We screened 174 MTC patients who underwent primary thyroid surgery at Samsung Medical Center between September 1998 and December 2019. Samsung Medical Center is a tertiary referral center in Seoul, Korea. To include pathologically confirmed primary tumors with a size of 2 cm or less confined to the thyroid gland (T1), we excluded 89 patients with primary tumor size greater than 2.0 cm (*n* = 44), and pathologically diagnosed with gross ETE (*n* = 2). We also excluded hereditary or familial MTC (*n* = 26) due to bilaterality, initial distant metastasis (*n* = 7), concurrent papillary thyroid carcinoma larger than 1.0 cm or papillary thyroid microcarcinoma with node metastasis (*n* = 6), and insufficient clinical information (*n* = 4). The rationale for choosing a primary tumor size of 2 cm as the cut-off is discussed in the “Discussion” section. After exclusion, 85 MTC patients were enrolled in this study. The need to obtain informed consent from the patients was explicitly waived by the Institutional Review Board of the Samsung Medical Center (IRB File No. 2020-07-007) which approved the protocols of this study. This study was performed in accordance with the committee’s guidelines.

### Surgical methods

The surgical strategy was performed according to the ATA guideline^11^ According to the guidelines, total thyroidectomy and prophylactic CND were performed if the patient was diagnosed with MTC preoperatively, regardless of the size of the primary tumor. Ipsilateral LN dissection or contralateral LN dissection was conducted when lateral lymph node metastasis was detected on preoperative imaging. Patients with negative lymph node metastases on preoperative imaging underwent prophylactic lateral lymph node dissection at the surgeon's discretion. The term "ipsilateral" refers to the same side as the primary tumor, and "contralateral" refers to the opposite side of the primary tumor. For bilateral tumors, the largest tumor was considered the primary tumor.

### Study design

Preoperative cervical lymph nodes were assessed using US and computed tomography (CT). Metastatic lymph nodes were considered when US features included round shape, presence of necrosis, presence of calcification, loss of the nodal hilum, and abnormal vascularity^39,40^. CT scans were performed preoperatively in patients with aggressive features on US scans or at the physician’s discretion. Metastatic LNs were considered when CT features included calcification, cystic or necrotic change, heterogeneous cortical enhancement, and strong enhancement without hilar vessel enhancement^41^. The US and CT results were reported level-by-level. Clinical staging of regional LN metastasis was performed if either US or CT features suggested malignancy. Based on these features, we classified patients into clinical N0 (cN0), clinical N1a (cN1a), and clinical N1b (cN1b) categories based on preoperative US and CT results using the 8th American Joint Committee on Cancer/Tumor-Node-Metastasis (AJCC/TNM) system.

Surgical specimens were examined by experienced pathologists, and histopathology results of LN status were reported level-by-level. We classified patients into pathological N0 (pN0), pathological N1a (pN1a), and pathological N1b (pN1b) categories using the 8th AJCC/TNM system. We classified patients who did not undergo node dissection as pathological Nx (pNx). We defined occult LN metastasis as the presence of a pathologically confirmed metastatic LN in the absence of suspicion of metastasis on preoperative US or CT.

Disease-free survival (DFS) was defined as the time from initial surgery to the date of the first structural recurrence or distant metastasis or to the date of the last censoring event. Structural recurrence of neck LN metastasis was confirmed histopathologically after fine-needle aspiration or surgery. Distant metastasis was detected by chest and/or abdominopelvic CT and/or 19-fluorodeoxyglucose positron emission tomography (FDG-PET) and/or was pathologically confirmed.

Our serum calcitonin level measurement method has been previously published^35^. In brief, we used immunoradiometric assay: MEDGENIX CT-U.S.-IRMA kit (BioSource Europe S.A., Belgium) from 1995 to 2005, and DSL-7700 ACTIVE IRMA kit (Diagnostic Systems Laboratories, Inc., Webster, TX) from 2005 to 2007. Since then it has been replaced by the current immunoradiometric assay (CT-US-IRMA, DIAsource ImmunoAssays SA, Louvain-la-Neuve, Belgium). The minimum detection limit was 0.9 pg/mL, and a serum calcitonin of < 5.0 pg/mL was considered within the normal range. Serum carcinoembryonic antigen (CEA) was measured using a radiometric assay using a commercial assay kit (CEA-RAICT, Cisbio biointernational, Gif-sur-Yvette, France), and 0–5 ng/mL was considered normal.

### Statistical analysis

Continuous variables are presented as mean ± standard deviation (SD) or median and interquartile range (IQR), as appropriate. Categorical variables are presented as numbers and percentages. The Kaplan–Meier analysis and the log-rank test were used to predict DFS. Multivariate Cox proportional hazards models were performed using backward elimination with a univariate inclusion criterion of P < 0.20 to assess the independent impact of covariates on disease-free survival. Statistical analysis was performed using SPSS version 25.0 for Windows (IBM, Chicago, IL, USA), and a P-value less than 0.05 was considered statistically significant.

### Supplementary Information

Supplementary Table 1.

## Data Availability

The datasets generated and/or analyzed during the current study are not publicly available but are available from the corresponding author on reasonable request.
