# Supplementary material for: Preoperative identification of low-risk medullary thyroid carcinoma: potential application to reduce total thyroidectomy
Source: Sci Rep. 2023 Sep 20;13:15663. doi: 10.1038/s41598-023-42907-3 (PMC10511442; doi:10.1038/s41598-023-42907-3)
Supplement: Supplementary file 1 — Supplementary Table 1. [file 41598_2023_42907_MOESM1_ESM.docx]

**Supplementary Material**

**Supplementary Table 1** Disease-free survival in groups categorized based on nodal staging and preoperative serum calcitonin level.

|  | No of patients (n) | No of  recurrence (n) | DFS  5-year (%) | DFS  10-year (%) |
| --- | --- | --- | --- | --- |
| Clinical category |  |  |  |  |
| cN0, CT ≤ 250 pg/mL | 45 | 3 | 94.7 | 90.2 |
| cN0, CT > 250 pg/mL | 19 | 1 | 100 | 92.3 |
| cN1, CT ≤ 250 pg/mL | 3 | 0 | - | - |
| cN1, CT > 250 pg/mL | 16 | 7 | 77.1 | 44.1 |
| Pathological category |  |  |  |  |
| pN0, CT ≤ 250 pg/mL | 41 | 2 | 96.6 | 91.5 |
| pN0, CT > 250 pg/mL | 12 | 0 | - | - |
| pN1, CT ≤ 250 pg/mL | 7 | 1 | 85.7 | 85.7 |
| pN1, CT > 250 pg/mL | 23 | 8 | 84.8 | 54.8 |

cN0, clinical N0; cN1, clinical N1; pN0, pathological N0; pN1, pathological N1; CT, calcitonin (pg/mL); No, number; DFS, disease-free survival.
